# Rear Admiral James Rufus Tryon

**Published:** 1912-04

**Authors:** 


Rear Admiral James Rufus Tryon, U.S.N., retired, but in charge of the Sailors' Snug Harbor on Staten Island, died March 21, 1912. Immediately after graduation, he entered the Navy as Assistant Surgeon, during the Civil War, and as indicated by his rank, rose to be Surgeon General.

